# POEMS syndrome complicated by portal hypertension resembling decompensated cirrhosis: a case report and diagnostic insights

**DOI:** 10.3389/fmed.2025.1654338

**Published:** 2025-11-03

**Authors:** Hua Zhou, Yangdong Zhou, Lifang Wu, Ling Yan, Jie Wei, Weixian Chen, Xi Huang, Shaocheng Zhang

**Affiliations:** ^1^Department of Laboratory Medicine, The Second Affiliated Hospital of Chongqing Medical University, Chongqing, China; ^2^Department of Clinical Laboratory, Zhuhai People's Hospital (Zhuhai Clinical Medical College of Jinan University), Zhuhai, China; ^3^Department of Hematology, The Second Affiliated Hospital of Chongqing Medical University, Chongqing, China; ^4^Department of Laboratory Medicine, The Second Affiliated Hospital of Chengdu Medical College (Nuclear Industry 416 Hospital), Chengdu, China; ^5^Western Institute of Digital-Intelligent Medicine, Chongqing, China

**Keywords:** POEMS syndrome, cirrhosis, portal hypertension, M-protein, autologous stem cell transplantation (ASCT)

## Abstract

A 39-year-old woman presented with progressive fatigue and abdominal distension over 6 months, accompanied by limb numbness in the last 3 months. She was initially diagnosed with decompensated cirrhosis at another hospital, with ascites and esophagogastric varices. Symptoms partially improved with diuretic therapy. However, 3 months later, she developed peripheral neuropathy characterized by “numbness in hands, lower legs, and feet, with a cotton–wool sensation while walking.” Further investigations at our hospital revealed immunoglobulin A (IgA)-λ type M-protein by immunofixation electrophoresis (IFE), elevated vascular endothelial growth factor (VEGF) (145.96 pg/mL), multiple lymphadenopathies, and endocrine abnormalities (hypothyroidism and menstrual irregularities), leading to a diagnosis of Polyneuropathy, Organomegaly, Endocrinopathy, M-protein, Skin changes (POEMS) syndrome. Following chemotherapy with the carfilzomi, pomalidomide, and dexamethasone (KPD) regimen and autologous hematopoietic stem cell transplantation (ASCT), the patient showed significant improvement in neurological function and systemic symptoms. This case highlights that after excluding common causes of cirrhosis, such as viral hepatitis, autoimmune liver disease, Wilson's disease, and metabolic dysfunction-associated steatohepatitis (MASH), the patient received repeated symptomatic treatment for cirrhosis. Furthermore, the cirrhotic facies resembled the skin hyperpigmentation of POEMS syndrome, contributing to atypical presentations and diagnostic delay. POEMS syndrome should be suspected in patients with unexplained cirrhosis, ascites, and multisystem damage. Immunofixation electrophoresis for monoclonal protein is crucial for definitive early diagnosis, and VEGF testing also holds certain diagnostic significance.

## 1 Introduction

Polyneuropathy, Organomegaly, Endocrinopathy, M-protein, Skin changes (POEMS) syndrome is a rare multisystem disorder associated with plasma cell dyscrasia, with an estimated annual incidence of approximately 0.3 per 100,000 (1). While its precise etiology and pathogenesis remain incompletely understood, the clinical manifestations predominantly include polyneuropathy, organomegaly, endocrinopathy, monoclonal immunoglobulinemia, and skin changes. The initial symptom is often insidious polyneuropathy, which may transiently respond to symptomatic treatment. The core pathological mechanisms involve clonal plasma cell proliferation and vascular endothelial growth factor (VEGF) overexpression (2). Classic features include demyelinating polyneuropathy (100%), hepatosplenomegaly (50–80%), endocrine dysfunction (67–84%), M-proteinemia (95%), and skin changes (68–89%) (3). We report a case of POEMS syndrome presenting initially as decompensated cirrhosis and discuss diagnostic and therapeutic strategies based on relevant literature.

## 2 Case report

### 2.1 Clinical presentation

A 39-year-old woman presented with insidious fatigue, abdominal distension, and left ear pain 6 months prior. External computed tomography (CT) portography (29 September 2024) revealed cirrhosis, splenomegaly, and minimal ascites. Gastroscopy confirmed esophagogastric varices. After excluding viral hepatitis [hepatitis B surface antigen (HBsAg) and hepatitis C virus antibody (anti-HCV) negative], autoimmune liver disease (negative autoantibody profile), and Wilson's disease (normal ceruloplasmin), a preliminary diagnosis of “cryptogenic decompensated cirrhosis” was made. Abdominal distension improved with diuretics and hepatoprotective agents. After 3 months, she developed progressive distal limb numbness and muscle weakness (muscle strength grade IV). Electromyography (EMG) (23 December 2024) showed slowed sensory conduction velocities in bilateral median, radial, tibial, and peroneal nerves.

### 2.2 Multidisciplinary diagnostic workup

#### 2.2.1 History summary

A 39-year-old woman complained of “fatigue for 6 months and progressively worsening limb numbness over 2 months.” Diagnosed with decompensated cirrhosis at another hospital on 29 September 2024, she showed no significant improvement after diuretics and hepatoprotective therapy. Symptoms markedly worsened 5 days before admission, limiting daily activities, causing walking difficulty, and impairing object handling. She was transferred to the Department of Infectious Diseases on 7 February 2025.

#### 2.2.2 Multidisciplinary evaluation

(I) Infectious Diseases: Cirrhosis with portal hypertension (Child-Pugh B) was confirmed, with viral and autoimmune causes ruled out. A liver biopsy was recommended to determine the etiology. The patient was advised to continue diuretics (spironolactone and furosemide) and hepatoprotection (silibinin).

(II) Neurology (Key Findings): Reduced muscle tone, absent tendon reflexes, muscle strength grade IV (distal > proximal), and impaired somatic sensation in limbs. Slowed nerve conduction velocities and reduced amplitudes on EMG. The diagnosis was “Cirrhosis with Peripheral Neuropathy,” with other causes such as immune, metabolic, and inflammatory causes.

(III) Nephrology: Diagnosed renal insufficiency (urea 8.02 mmol/L ↑, creatinine 97.2 μmol/L ↑, uric Acid 583.4 μmol/L ↑, creatinine clearance 58.5 mL/min ↓, and estimated glomerular filtration rate (eGFR) 63.5 mL/min ↓). To exclude primary/secondary causes, it has been recommended to conduct parathyroid hormone (PTH), antinuclear antibody (ANA), extractable nuclear antigens (ENA), including anti-double-stranded DNA (anti-dsDNA), vasculitis antibody panel, and renal ultrasound tests.

(IV) Rheumatology/Immunology: Elevated immunoglobulin A (IgA, 4.44 g/L ↑), decreased complement 3 (C3, 0.063 g/L ↓), and reduced total lymphocyte count (1,435.07/μL ↓), and significant generalized skin hyperpigmentation.

(V) Hematology (Suspicion Raised): Based on the combination of limb numbness and weakness, abnormal nerve conduction, unexplained cirrhosis/splenomegaly, ascites and pleural effusion, menstrual irregularity, and elevated IgA levels, POEMS syndrome was suspected., A VEGF assay, sex hormones, serum immunofixation electrophoresis, superficial lymph node ultrasound, and bone marrow examination (aspiration/biopsy/flow cytometry for clonal plasma cells) were recommended.

(VI) Endocrinology: Abnormal cortisol rhythm (4 p.m.: 172.50 nmol/L, 0 a.m.: 181.10 nmol/L, and 8 a.m.: 304.50 nmol/L), elevated ACTH (adrenocorticotropic hormone, 76.20 pg/mL ↑), low cortisol (28.35 nmol/L ↓), and elevated TSH (thyroid-stimulating hormone, 13.400 μIU/mL ↑). Patient reported amenorrhea for 3 months. It was diagnosed as subclinical hypothyroidism.

(VII) Hematology (Confirmation): VEGF elevated (145.96 pg/mL). Serum immunofixation electrophoresis (19 February 2025): IgA positive ↑, IgG positive ↑, IgM negative, κ negative, and λ positive ↑. Ultrasound showed bilateral cervical, axillary, and inguinal lymphadenopathy. Ascites (mild-moderate) suggested on ultrasound. Positron emission tomography/CT (PET/CT) ordered for systemic tumor assessment.

#### 2.2.3 Key diagnostic evidence

(I) Bone Marrow Biopsy: Normocellular marrow, easily identifiable plasma cells among granulocytic, erythroid, and megakaryocytic lineages.

(II) PET/CT: ① Multiple enlarged lymph nodes (bilateral neck I–V, axillae, hila, mediastinum, abdomen, retroperitoneum, and iliac vessels) with mildly increased fluorodeoxyglucose (FDG) uptake and variable C-X-C chemokine receptor type 4 (CXCR4) expression; ② Splenomegaly with diffuse CXCR4 expression, normal FDG uptake; ③ Diffusely increased FDG uptake and CXCR4 expression in bones, suggesting hypercellular marrow; ④ Peritonitis, scattered ascites; ⑤ Subcutaneous edema in neck/chest/abdomen/pelvis/bilateral thighs/buttocks.

(III) Flow Cytometry: Abnormal plasma cells accounted for 0.54% of nucleated cells in bone marrow.

#### 2.2.4 Diagnostic criteria

(I) Major: Polyneuropathy (EMG confirmed).

(II) Minor: Monoclonal plasma cell proliferation (serum IgA/λ+, bone marrow cytological abnormalities); extravascular volume overload (ascites, pleural effusion, and edema); endocrinopathy (hypothyroidism and adrenal axis dysfunction); skin changes (hyperpigmentation); serum VEGF was normal but close to the upper limit of the reference range; and organomegaly (splenomegaly).

### 2.3 Treatment and outcome

#### 2.3.1 Treatment

Induction therapy with KPD regimen (carfilzomib 20/36 mg/m^2^ days 1, 8, and 15; pomalidomide 4 mg days 1–21; and dexamethasone 20 mg weekly) was performed in 2 cycles; successful CD34^+^ cell mobilization and collection (3.9 × 106/kg) were performed; and conditioning with melphalan (140 mg/m^2^) was carried out.

#### 2.3.2 Autologous stem cell transplant (ASCT)

Performed on 14 April 2025. Neutrophil engraftment [absolute neutrophil count (ANC), >0.5 × 10^9^/L] was achieved on day +4 (ANC, 1.86 × 10^9^/L). Platelet engraftment (20 × 10^9^/L without transfusion) was achieved.

#### 2.3.3 Outcome

On 16 June 2025, the blood routine showed good recovery of hematopoietic function. The patient reported improved numbness, could walk independently, and was able to carry slightly heavier objects on her own. She could also shake hands with the doctor, who observed significant improvement in muscle strength.

**Figure 1:**
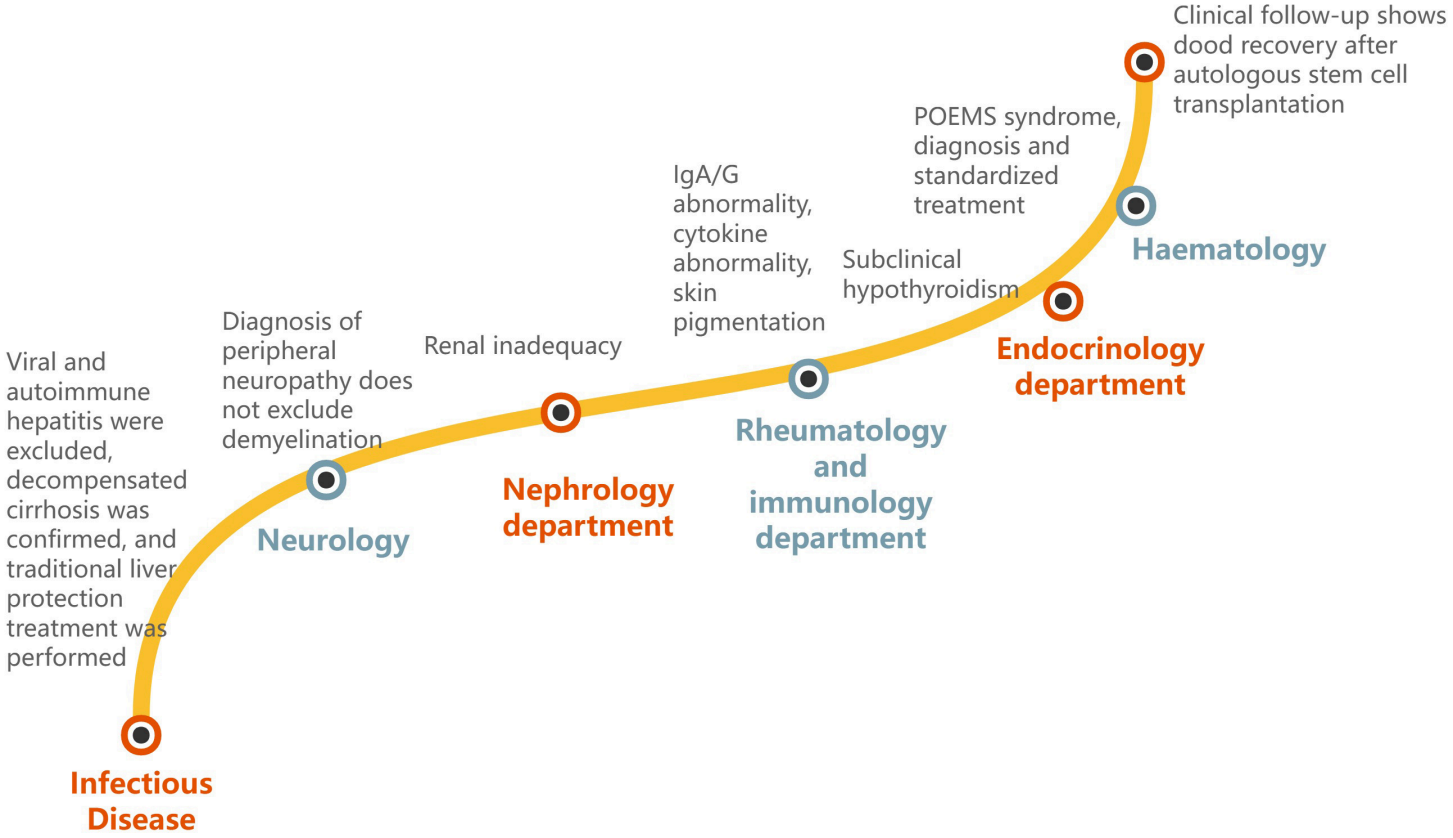
Schematic diagram of multidepartment collaboration of patients.

The brief diagnostic process of the above process is shown in Figure 1. The results of the positive tests that are meaningful in the diagnosis process are shown in Table 1, and those that are negative for exclusion are shown in Table 2. Changes in immunofixation electrophoresis, hematology tests, CT scans, VEGF, and other examinations before and after treatment indicate that the treatment is effective, as shown in Tables 3–7, respectively.

**Table 1:** Key positive laboratory findings supporting diagnosis.

**Laboratory test**	**Results (status)**	**Reference range**
High-sensitivity C-reactive protein (hs-CRP)	>5.0 mg/L ↑	0–5.0
Tumor necrosis factor-alpha (TNF-α)	9.50 ↑/mL	0–4.5
**Liver function**		
Alkaline phosphatase (ALP)	133 ↑U/L	35–100
Gamma-glutamyl transferase (GGT)	54 ↑U/L	7–45
Prealbumin	126 ↓g/L	150–380
**Renal function**		
Urea	11.32 ↑mmol/L	2.60–7.50
Creatinine	90.4 ↑μmol/L	41.0–73.0
Uric acid	561.9 ↑μmol/L	150.0–360.0
Creatinine clearance (Ccr)	63.6 ↓mL/min	75.0–300
Estimated GFR (eGFR)	69.4 ↓mL/min	80.0–300
**Lipids**		
HDL cholesterol	0.64 ↓mmol/L	1.29–1.55
Apolipoprotein A1	0.66 ↓g/L	1.0–1.60
**Immunoglobulins**		
Immunoglobulin G (IgG)	16.30 ↑g/L	7.51–15.60
Immunoglobulin A (IgA)	4.44 ↑g/L	0.70–4.00
Free light chain (κ)	33.4 ↑mg/L	6.7–22.4
Free light chain (λ)	153.0 ↑mg/L	8.3–27.0
**Electrolytes/metabolites**		
Phosphorus	1.62 ↑mmol/L	0.85–1.51
Lactate	4.23 ↑mmol/L	1.32–2.90
**Coagulation function**		
Prothrombin time (PT)	15.0 ↑S	11.0–14.5
Activated partial thromboplastin Time (APTT)	45.5 ↑S	28.0–44.0
APTT ratio	1.34 ↑S	0.80–1.30
Erythrocyte sedimentation rate (ESR)	36 ↑mm/h	2–20
**Lymphocyte count**		
Total lymphocyte count FS/SS	1435.07 ↓/ul	1530.00–3700.00
**Vitamins**		
Erythrocyte folate	1415.34 ↑ng/mL	140.00–836.00
Vitamin B6 (Phosphopyridoxal)	2.49 ↓ng/mL	5.00–50.00
**Thyroid function**		
Thyroid-stimulating hormone (TSH)	13.400 ↑μIU/mL	Non-pregnant 0.35–5.00
**Endocrine function**		
Plasma cortisol (8 a.m)	28.35 ↓nmol/L	
Adrenocorticotropic hormone (ACTH)	76.20 ↑pg/mL	4.70–48.80
Glycated hemoglobin (HbA1c)	3.7 ↓%	4.0–6.0
**Bone marrow**		
Flow cytometry (abnormal plasma cells)	Plasmacyte 0.54% of NC ↑	
Morphology	Hypercellular, active plasmacytosis	
Hypercellular, active plasmacytosis	Neutrophils accounted for 61%, mainly phagocytic granulocytes, with normal morphology; eosinophils were observed in 2%; mature red blood cells were not abnormal; scattered platelets were observed; lymphocytes accounted for 31%, with normal morphology; monocytes accounted for 6%; no parasites were observed	
**Imaging studies**		
PET/CT:	① Multiple enlarged lymph nodes in bilateral neck I–V area, bilateral axilla, bilateral hilar region, mediastinum, abdominal cavity, retroperitoneum, and bilateral iliac vascular pathway area, mild increase in FDG metabolism, increased expression of CXCR4 to varying degrees; multiple enlarged lymph nodes ② Enlarged spleen, CXCR4 expression increased diffusely	
Thorax CT	Multiple enlarged lymph nodes were found in the right heart diaphragmatic angle, intra-abdomen, and retroperitoneum, which were larger than the anterior part; bone density was increased in the sternum, some vertebrae and pelvis, and bilateral femur; a small amount of fluid was found in both pleural cavities, and scattered inflammation was detected in both lungs	
Abdomen CT	Multiple enlarged lymph nodes were found in the right heart diaphragmatic angle, intra-abdomen, and retroperitoneum, which were larger than the anterior part; bone density was increased in the sternum, some vertebrae and pelvis, and bilateral femur; a small amount of fluid was found in both pleural cavities, and scattered inflammation was detected in both lungs. Multiple enlarged lymph nodes were found in the right heart diaphragmatic angle, intra-abdomen, and retroperitoneum, which were larger than the anterior part; bone density was increased in the sternum, some vertebrae and pelvis, and bilateral femur; a small amount of fluid was found in both pleural cavities, and scattered inflammation was detected in both lungs	
Type-B ultrasonic	Enlarged lymph nodes in the bilateral neck, armpit, and inguinal regions	
Abdominal ultrasound	Small to moderate amount of fluid in the abdominal cavity	
Chest ultrasound	pleural effusion	
Urological B-ultrasound	Renal urinary salt crystallization	
Portal computed tomography venography (CTV)	Manifestations of cirrhosis, splenomegaly, a small amount of ascites, and portal hypertension	
Electromyogram nerve conduction velocity (NCV)	The conduction velocity of the bilateral median nerve, radial nerve, tibial nerve, and peroneal nerve is slowed down.	
Magnetic resonance imaging (MRI)	High signal of white matter in the brain (Fazekas grade I) suggests small vessel-related lesions; subcutaneous nodules in the bilateral occipital regions.	
**Serum fixation electrophoresis**		
IgA	Positive	
IgG	Positive	
λ	Positive	
Vascular endothelial growth factor (VEGF)	145.96 pg/mL	0.00–160.00 pg/mL
Dehydroepiandrosterone sulfate	65.48 ↓	74.80–410.20 μg/dl
Prolactin	41.20 ↑	Follicular phase 6.2–23.4; Ovulatory period 6.2–23.4; Luteal phase 6.2–23.4; Menopause 4.2–18.4 μg/L

**Table 2:** Key negative laboratory findings.

**Laboratory tests**	**Status**	**Reference**
Hepatitis B surface antigen (HBsAg)	Neg 0.000 IU/mL	0.000–0.050
Hepatitis B surface antibody (HBsAb)	2.850 mIU/mL	0.000–10.000
Hepatitis B virus e antigen (HBeAg)	0.350 S/co	0.000–1.000
Hepatitis B e antibody	1.750 S/co	1.000–999.000
Hepatitis B core antibody	0.200 S/co	0.000–1.000
Alpha-fetoprotein (AFP)	2.93 ng/mL	0.00–13.20
Hepatitis C antibody (anti-HCV)	0.266 S/co	0.000–1.000
Hepatitis C core antigen	0.133 S/co	0.000–1.000
Vitamin B12	238.00 pg/mL	180.00–914.00
Anti-cyclic citrullinated peptide (CCP) antibody	2.07 RU/mL	0.00–20.00
Anti-endothelial cell antibody (AECA)	Negative	Negative
Cytoplasmic anti-neutrophil cytoplasmic antigen (cANCA)	Negative	Negative
Anti-perinuclear anti-neutrophil cytoplasmic antibody (pANCA)	Negative	Negative
Atypical anti-neutrophil cytoplasmic antigen (ANCA)	Negative	Negative
Anti-proteinase 3 (PR3)	1.4	
Anti-myeloperoxidase (MPO)	1.3	
Anti-dsDNA antibody	2.7 IU/mL	0–30.0
Anti-C1q antibody	< 4.0 RU/mL	0.0–20.0
Anti-nuclear antibody spectrum		
Anti-dsDNA	< 10 IU/ml	0.0–100.0
Anti-u1-snRNP (U1-small nuclear ribonucleoprotein)	Negative 0.1 AI	Negative < 10
Anti-Sm (Smith antibody)	Negative 0.1 AI	Negative < 10
Anti-Sjögren's syndrome A antigen or Robert antigen (SSA/Ro52)	Negative 0.8 AI	Negative < 10
Anti-SSA/Ro60	Negative 0.3 AI	Negative < 10
Anti-Sjögren's syndrome B antigen or lane antigen (SSB/La)	Negative 0.1 AI	Negative < 10
Anti-proliferating cell nuclear antigen (PCNA)	Negative 0.4 AI	Negative < 10
Total protein (TP)	70.0 g/L	65.0–85.0
Albumin (ALB)	40.6	40.0–55.0
Alanine aminotransferase (ALT)	12 U/L	7–40
Aspartate amino transferase (AST)	13 U/L	13–35
Total bilirubin (TBIL)	14.6 μmol/L	5.1–28
Conjugated bilirubin (DB)	4.5 μmol/L	0–10.0
Anti-streptolysin (ASO)	48.20	0.0–408.00 IU/ml
Rheumatoid factor (RF)	< 20.0 IU/ml	0–20.0

**Table 3:** Immunofixation electrophoresis results of patients.

**(A) Immunofixation electrophoresis results: IgAλ type (19 February 2025)**	**(B) Immunofixation electrophoresis results: IgAλ type (28 March 2025)**	**(C) Immunofixation electrophoresis results: IgAλ type (18 June 2025) (weakly positive)**
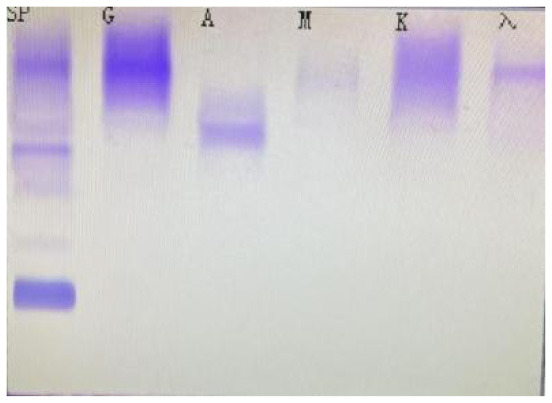	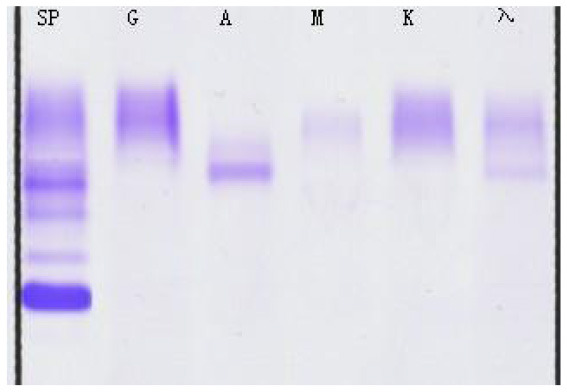	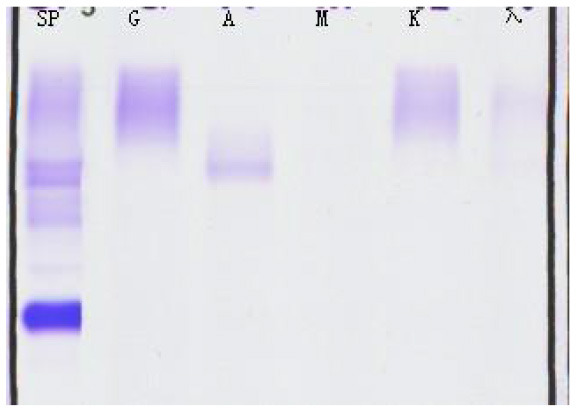

From A to C, the IgA and λ bands observed in serum immunofixation electrophoresis are becoming progressively weaker, with the λ band even disappearing, indicating that the levels of IgA and λ in the patient's serum are decreasing, suggesting that the treatment is effective. (GAM, IgG, IgA, and IgM; G, IgG; A, IgA; M, IgM; D, IgD; E, IgE; κ, κ chain; λ, λ chain; κf, free κ chain; λf, free λ chain).

**Table 4:** Comparison of blood routine results of patients, suggesting neutrophils are implanted successfully.

**No**.	**Item**	**Results (8 February, 2025)**	**Results (17 April 2025) 3 days after ASCT**	**Results (27 April 2025) 7 days after ASCT**	**Results (5 May 2025) 14 days after ASCT**	**Results (12 May 2025) 21 days after ASCT**	**Results (16 June 2025) 35 days after ASCT**	**Unit**	**Reference value**
1	RBC	3.95	1.86 ↓	1.68 ↓	2.38 ↓	1.96 ↓	2.68	10^12^/L	3.80–5.10
2	HGB	121	58 ↓	51 ↓	73 ↓	61 ↓	91	g/L	115–150
3	HCT	36.5	17.4 ↓	15.3 ↓	22.8 ↓	19.5 ↓	27.2	%	35.0–45.0
4	MCV	92.5	93.4	91.1	95.8	99.5	101.4	fL	82.0–100.0
5	MCH	30.5	31.1	30.4	30.7	31.1	33.9	pg	27.0–34.0
6	MCHC	332	333	333	320	313	335	g/L	316–354
7	RDW	13.9	16.1 ↑	13.5	16.7↑	17.9	12.5	%	11.5–14.5
8	RDW-SD	46.6 ↑	53.1 ↑	44.4	56.5↑	63.9	48.3	fL	39.0–46.0
9	WBC	6.94	1.90 ↓	0.41 ↓	1.83↓	1.52↓	5.84	10^9^/L	3.50–9.50
10	NEU%	68.0	97.5 ↑	48.8	53.7	53.4	34.1	%	45.0–75.0
11	LYMPH%	23.0	1.1 ↓	26.8	29.5	28.9	57.5	%	20.0–50.0
12	MON%-M	6.2	0.0 ↓	24.4 ↑	15.8 ↑	16.4	5.7	%	3.0-−10.0
13	EOS%	2.3	0.7	0.0 ↓	0.5	1.3	2.5	%	0.4-−0.0
14	BAS%	0.5	0.7	0.0	0.5	0.0	0.2	%	0.0-−1.0
15	NEU#	4.72	1.86	0.20 ↓	0.98↓	0.81↓	1.99	10^9^/L	1.80–6.30
16	LYMPH#	1.60	0.02 ↓	0.11 ↓	0.54↓	0.44↓	3.36	10^9^/L	1.10–3.20
17	MON#	0.43	0.00 ↓	0.10	0.29	0.25	0.33	10^9^/L	0.10–0.60
18	EOS#	0.16	0.01 ↓	0.00 ↓	0.01↓	0.02	0.15	10^9^/L	0.02–0.52
19	BAS#	0.03	0.01	0.00	0.01	0.00	0.01	10^9^/L	0.00–0.06
20	PLT	185	50 ↓	11 ↓	45↓	42↓	82	10^9^/L	100–300
21	PDW	16.9	16.0	12.0	12.0	12.2	16.9		15.0–17.0
22	MPV	8.6	7.4 ↓	11.9	11.3	10.9	9.1	fL	7.6–13.2
23	PCT	0.159	0.037 ↓	0.010 ↓	0.050↓	0.050↓	0.075	%	0.108–0.282
24	Platelet ratio	18.8	10.4 ↓	38.4	35.8	30.2	21.0	%	13.0–43.0

ASCT, autologous stem cell transfer; #, absolute value. RBC: Red Blood Cell Count; HGB: Hemoglobin; HCT: Hematocrit; MCV: Mean Corpuscular Volume; MCH: Mean Corpuscular Hemoglobin; MCHC: Mean Corpuscular Hemoglobin Concentration; RDW: Red Cell Distribution Width; RDW-SD: Red Cell Distribution Width - Standard Deviation; WBC: White Blood Cell Count; NEU%: Neutrophil Percentage; LYMPH%: Lymphocyte Percentage; MONO%: Monocyte Percentage; EOS%: Eosinophil Percentage; BAS%: Basophil Percentage; NEU#: Neutrophil Count; LYMPH#: Lymphocyte Count; MONO#: Monocyte Count; EOS#: Eosinophil Count; BAS#: Basophil Count; PLT: Platelet Count; PDW: Platelet Distribution Width; MPV: Mean Platelet Volume; PCT: Plateletcrit; P-LCR: Platelet Larger Cell Ratio.

**Table 5:** CT images of the patient's abdomen.

**(A) Abdominal CT at admission (7 February 2025)**	**(B) Abdominal CT after treatment (27 April 2025)**
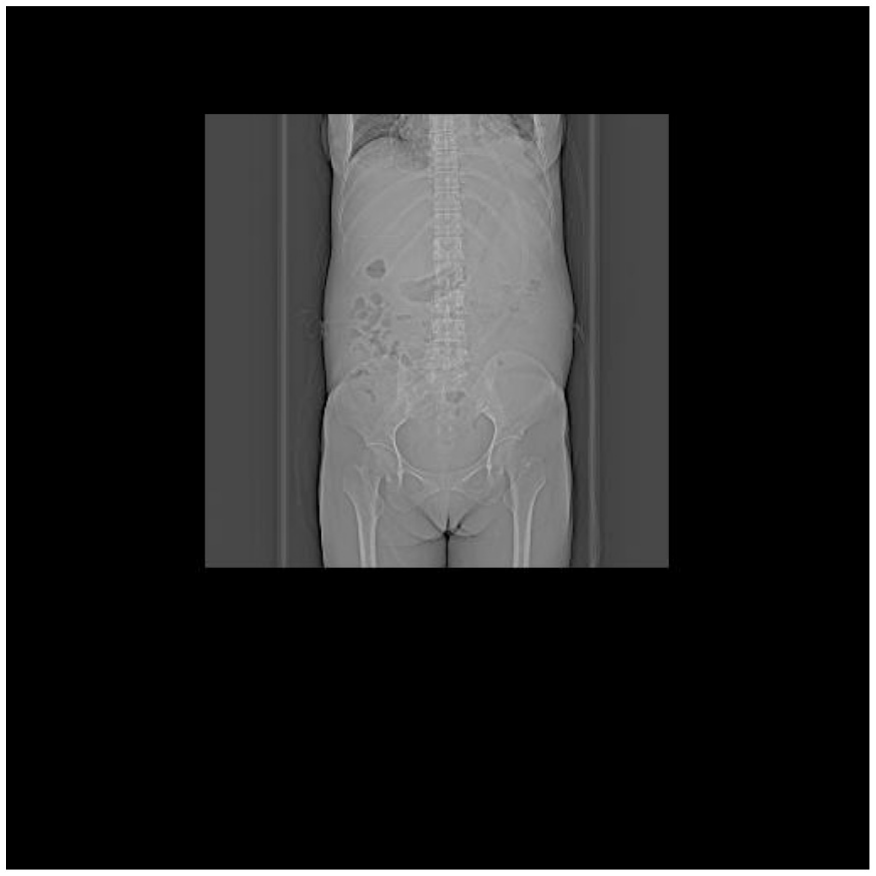	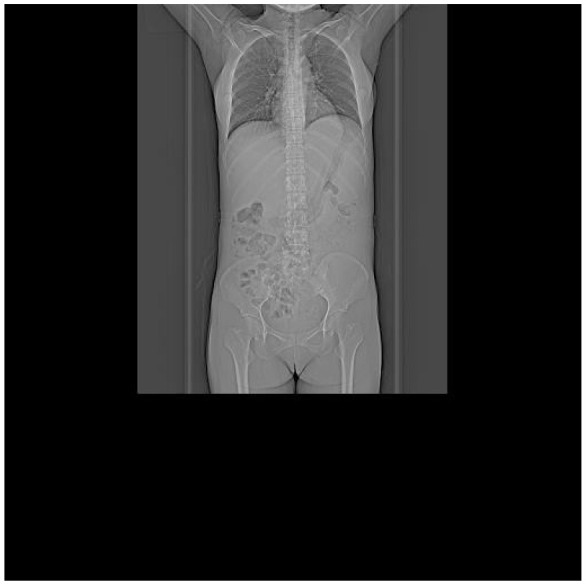
**(C) PET-CT at admission (25 February 2025)**	**(D) PET-CT at admission (25 February 2025)**
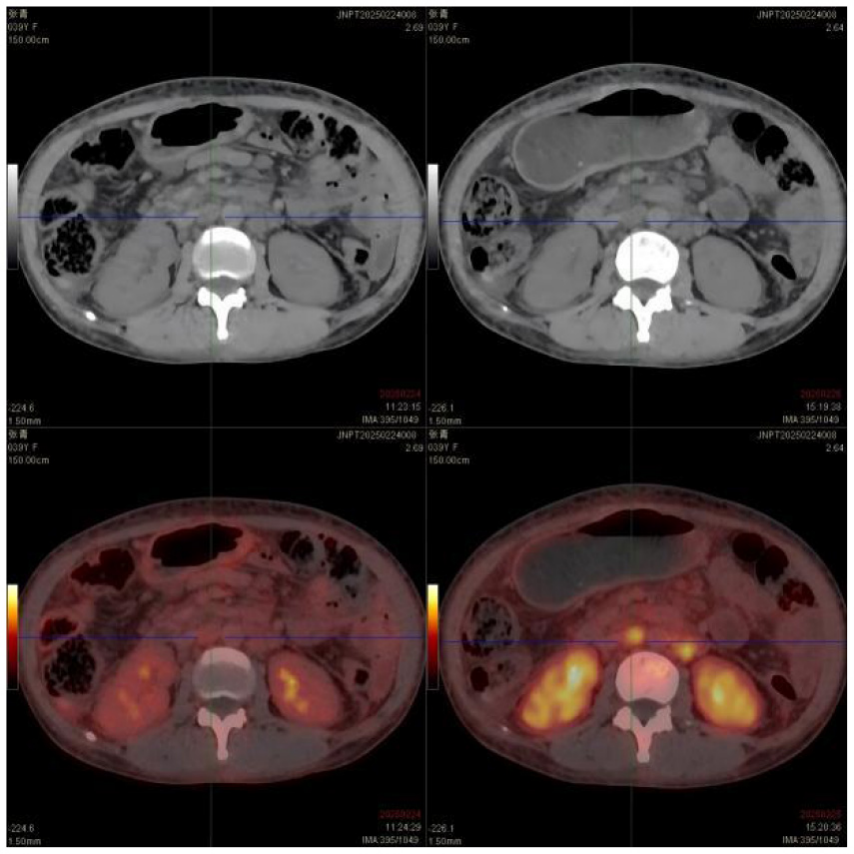	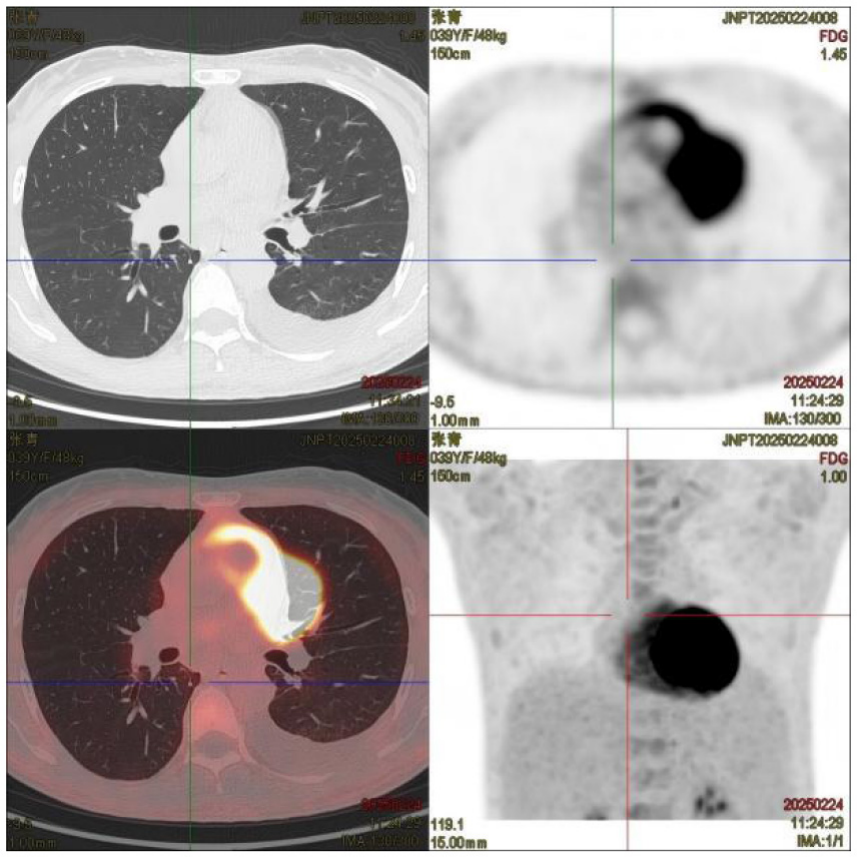
**(E) PET-CT at admission (25 February 2025), cervical vertebrae**	**(F) Abdominal CT after treatment (27 April 2025)**
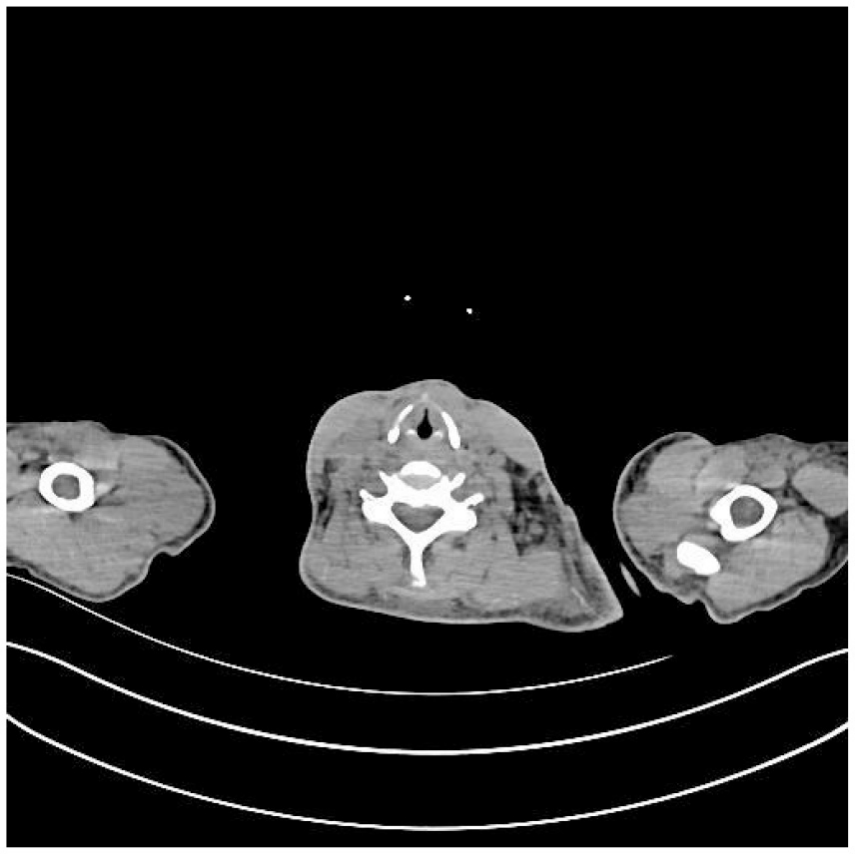	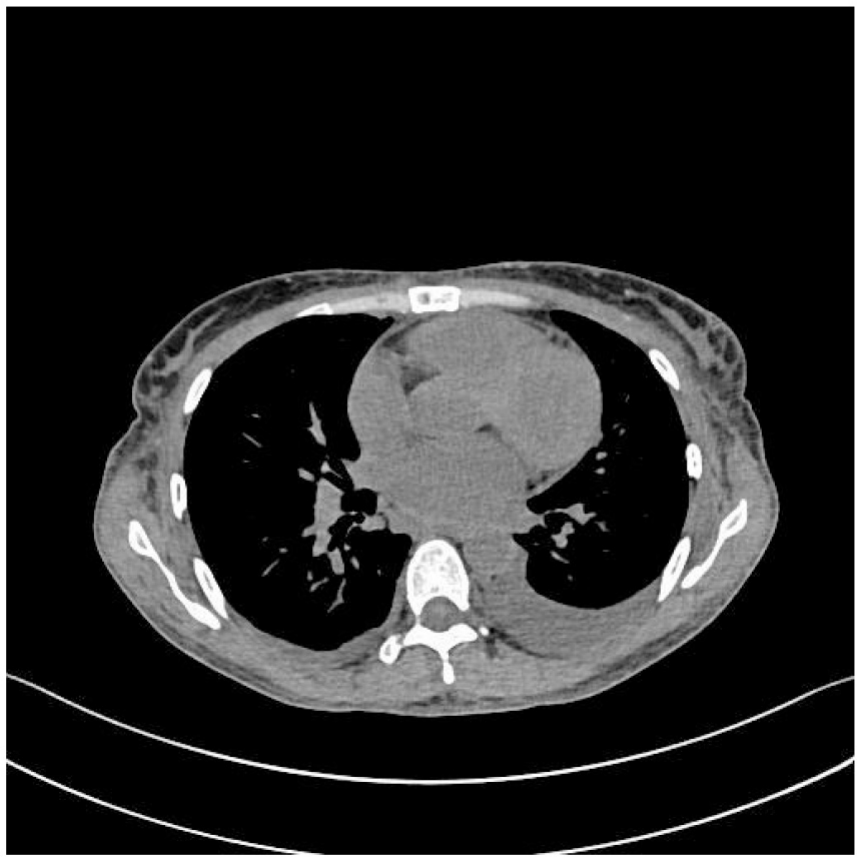

A and B are abdominal CT scan images before and after autologous stem cell transplantation. A shows a significant amount of ascites, while B indicates a marked reduction in ascites. C, D, and E are PET/CT images before autologous stem cell transplantation, which show increased bone density in multiple areas, elevated FDG metabolism, no abnormal increase in CXCR4 expression; splenomegaly with diffusely increased CXCR4 expression and no abnormal FDG metabolism; and inflammatory changes in the peritoneum with scattered effusions in the abdomen and pelvis. F shows improvement after treatment.

**Table 6:** Changes in VEGF before and after treatment.

**Item**	**17 February 2025 (Pre-autologous stem cell transplantation)**	**28 March 2025 (14 days after autologous stem cell transplantation)**	**7 April 2025 (24 days after autologous stem cell transplantation)**
VEGF ^pg/mL^	145.96	123.85	115.33

**Table 7:** Other main changes before and after treatment.

**Item**	**Before ASCT**	**After ASCT**
*Parazacco spilurus* subsp. spilurus-type plasma cell proportion by Bone marrow flow cytometry	0.54%	0.02%
Plasma cells accounting by bone marrow cytology examination	3%	2%
Immunoglobulin A (IgA)	4.44 g/L	2.42 g/L
Serum free light chain (λ)	153.0 mg/L	141.0 mg/L

## 3 Discussion

This patient was initially diagnosed with cirrhosis and portal hypertension. The subsequent development of peripheral neuropathy and renal insufficiency prompted immunofixation electrophoresis, which revealed M-proteinemia. Bone marrow pathology and endocrine evaluations ultimately confirmed the diagnosis of POEMS syndrome.

### 3.1 Differential diagnosis of peripheral neuropathy

Guillain–Barré Syndrome (GBS): Both GBS and POEMS can cause motor paralysis. However, GBS lacks associated organomegaly, endocrinopathy, skin changes, bone lesions, and elevated VEGF, all present in this case.

Chronic Inflammatory Demyelinating Polyneuropathy (CIDP): CIDP, an immune-mediated neuropathy, presents with symmetric limb weakness, sensory disturbances, and chronic progression/relapse. EMG shows demyelination, and CSF typically has elevated protein. It often responds to immunotherapy. POEMS and CIDP both affect motor/sensory nerves and slow conduction. However, CIDP does not feature organomegaly, endocrinopathy (thyroid), M-protein, skin changes, bone lesions, or elevated VEGF, all evident here. The constellation of polyneuropathy, lymphadenopathy, splenomegaly, elevated VEGF, skin hyperpigmentation, and M-protein confirmed POEMS.

### 3.2 Pathogenesis of liver injury

The uniqueness of this case lies in the initial presentation as cirrhosis and portal hypertension. A potential mechanism involves VEGF-mediated injury to hepatic sinusoidal endothelial cells. Studies suggest VEGF activates MMP-9, leading to degradation of the sinusoidal basement membrane, sinusoidal capillarization, and increased portal resistance (4).

VEGF activates MMP-9: Through pathways such as hypoxia-inducible factor (HIF-1α), VEGF increases the expression of MMP-9, particularly in the tumor microenvironment. MMP-9 degrades the basement membrane: MMP-9 specifically hydrolyzes type IV collagen, a key component of the hepatic sinusoidal basement membrane, disrupting the endothelial structure of the hepatic sinusoids. Hepatic sinusoidal capillaryization: After the basement membrane is degraded, the endothelium of the hepatic sinusoids loses its fenestrated structure and transforms into capillary-like vessels covered by a continuous basement membrane, leading to “capillaryization“ of the hepatic sinusoids. Increased portal vein resistance: The capillaryization of the hepatic sinusoids reduces the blood exchange area, increases blood flow resistance, and ultimately raises the pressure in the portal vein. In addition, the differential diagnosis between liver cirrhosis and portal hypertension should be paid more attention (5).

### 3.3 Optimizing diagnostic strategy

The median diagnostic delay for POEMS syndrome is 13 months (6); this case took 9 months. We propose a three-step diagnostic algorithm for patients with “cirrhosis + neuropathy”:

Serum Protein Studies: Serum protein electrophoresis + Immunofixation electrophoresis + Serum immunoglobulins + Serum free light chains (κ/λ ratio).

The VEGF assay is a critical diagnostic marker, with a cut-off of ≥200 pg/mL demonstrating a specificity of 95% and a sensitivity of 68% (7).

Systemic Imaging: PET/CT (sensitivity usually over 90% for detecting plasmacytomas). Bone marrow aspiration/biopsy with morphology and flow cytometry is essential for confirmation in suspected cases. PET/CT was able to detect MM osteolytic lesions with a sensitivity of approximately 80–90% and a specificity of 80–100% (8).

### 3.4 Treatment advances

ASCT has emerged as a potentially curative treatment for POEMS, improving short-term outcomes and long-term survival:

Symptom Improvement: Multiple studies show ASCT significantly improves polyneuropathy, skin changes, edema, and other systemic manifestations (9).

Hematologic Response: Although M-protein negativity is less common post-ASCT, significant reductions in VEGF levels correlate with neurologic recovery. Data from Peking Union Medical College Hospital shows a hematologic complete response (CR H) rate of 57.3% and a 5-year progression-free survival (PFS) of 72.2% post-ASCT (10). Large retrospective studies indicate 5-year overall survival (OS) rates of 89–92.8% for ASCT recipients, significantly higher than conventional chemotherapy.

In addition, we have summarized and compared the similarities and differences between some reported cases of portal hypertension and POEMS syndrome, as well as this case (Table 8).

**Table 8:** Reports on POEMS with portal hypertension and their comparisons with this case.

**Title of literature**	**Publication time**	**Patient basic information**	**Primary symptoms**	**Key findings**	**Main treat**	**Outcome**	**Similar to this article**	**Differences from this article**
A POEMS syndrome patient with idiopathic non-cirrhotic portal hypertension received the transjugular intrahepatic Portosystemic shunt: a case report and literature review(11)	2022	Male, 62-year old	Symptoms of black stool for 10 days	Transjugular intrahepatic portosystemic shunt (TIPS)	TIPS and venous embolization	Hepatic encephalopathy, which eventually leads to death	Portal hypertension	Therapeutic regimen
Case report: POEMS syndrome with portal hypertension (12)	2024	Male, 70-year old	Bloating, difficulty breathing	Pathological biopsy confirmed non-cirrhotic portal hypertension of unknown etiology	TIPS: Two courses of bortezomib combined with dexamethasone after treatment,	The patient died of systemic infection	Chemotherapy programme	Concurrent infection
POEMS syndrome and idiopathic portal hypertension: a possible association (13)	2017	Female, 48-year old	Upper gastrointestinal bleeding	Endoscopy revealed massive varices in the esophagus	Under a band ligation program, with beta-blocker, diuretics, and prophylactic anticoagulation	The patient remains stable	Gastric esophageal varices, middle-aged woman	β-blockers
Portal hypertension as the initial manifestation of POEMS syndrome: a case report (14)	2017	Male, 46 years old	Electromyography showed peripheral neuropathy	The immunofixation reveals monoclonal immunoglobulin A lambda protein	Lenalidomide in combination with or without dexamethasone	Effective treatment	Chemotherapy regimen; Immunofixation electrophoresis	No autologous stem cell transplantation
Polyneuropathy, Organomegaly, Endocrinopathy, M-protein, and Skin Changes (POEMS) Syndrome and Idiopathic Portal Hypertension: a rare association (15)	2022	Male, 46 years old	Postprandial vomiting and left tinnitus, accompanied by loss of appetite, weight loss, and excessive sweating	Abdominal examination reveals splenomegaly	Six courses of cyclophosphamide, thalidomide, and dexamethasone (CTD) regimens followed by autologous stem cell transplantation (ASCT)	The patient's condition improved significantly	Splenomegaly	No esophageal varices
POEMS syndrome with idiopathic portal hypertension: autopsy case and review of the literature (16)	2009	Female, 38 years old	Multiple neuropathies and edema	Enlarged spleen and papilledema	Decade-long corticosteroid therapy	Died at the age of 58 due to hepatic encephalopathy	Women of similar age	Autopsy showed typical portal vein fibrosis and small branch occlusion in the liver
The characteristics of ascites in patients with POEMS syndrome (17)	2013	106 cases of POEMS syndrome	Assessment of the presence of ascites	Levels of complement, cytokines, and clinical chemistry parameters in peripheral blood and ascites samples	/	/	Ascites	Non-portal hypertensive caused by low SAAG
Portal hypertension and neutrocytic ascites in POEMS syndrome (18)	1998	Male, 52 years old	Portal hypertension and neutropic ascites	A sterile neutrophilic ascites	/	/	Ascites	First description of sterile neutrophilic ascites
Reversible pulmonary hypertension in POEMS syndrome–another etiology of triggered pulmonary vasculopathy? (19)	2000	/	With precapillary pulmonary hypertension and Raynaud phenomenon	Pulmonary artery hypertension	Short-term corticosteroid and long-term nifedipine treatment responded well	The patient's condition has improved significantly	Portal hypertension	pulmonary artery hypertension
Porto-sinusoidal vascular disorder, report of a novel association with POEMS syndrome. Future challenge for the hepatologist (20)	2023	Male, 59 years old	Porto-Sinusoidal Vascular Disorder (PSVD)	Refractory ascites, pleural effusion, and high-risk varicose veins	Treated with dexamethasone and lenalidomide	The patient's condition has improved significantly	Chemotherapy programme	Meet the diagnostic criteria for PSVD

### 3.5 Future research directions

Biomarker-Guided Therapy: Dynamic VEGF monitoring serves as an efficacy indicator. 18F-FDG PET/CT assesses early metabolic response. Associations with Vitamin B6/B12 deficiency warrant nutritional screening.

Novel Conditioning Regimens: Exploring targeted agents (e.g., Daratumumab) combined with ASCT, or developing lower-toxicity regimens to improve tolerability in older patients.

Long-Term Follow-up and Mechanistic Studies: Further elucidation of post-ASCT immune reconstitution, neural repair mechanisms, and risk factors for relapse is needed.

## 4 Conclusion

This case illustrates that POEMS syndrome can manifest as an atypical liver disease process. Clinicians must maintain a high index of suspicion for this “diagnostic puzzle,” particularly when common causes of cirrhosis are excluded. The 9-month diagnostic delay highlights the need for early implementation of the proposed algorithm incorporating immunofixation, VEGF, and PET/CT. It is recommended to include immunofixation electrophoresis in the routine evaluation of unexplained cirrhosis, especially in the presence of neurological or endocrine abnormalities. Early ASCT significantly improves prognosis, and a multidisciplinary approach is paramount for timely diagnosis and effective management.

## Data Availability

The raw data supporting the conclusions of this article will be made available by the authors, without undue reservation.

## References

[1] Bardwick PA Zvaifler NJ Gill GN Newman D Greenway GD Resnick DL. Plasma cell dyscrasia with polyneuropathy, organomegaly, endocrinopathy, M protein, and skin changes: the POEMS syndrome Report on two cases and a review of the literature. Medicine (Baltimore). (1980) 59:311–22. 10.1097/00005792-198007000-00006 6248720

[2] Dispenzieri A. POEMS syndrome: update on diagnosis, risk-stratification, and management. Am J Hematol. (2021) 96:872–88. 10.1002/ajh.26240 34000085

[3] Dispenzieri A Kyle RA Lacy MQ Rajkumar SV Therneau TM Larson DR. POEMS syndrome: definitions and long-term outcome. Blood. (2003) 101:2496–506. 10.1182/blood-2002-07-2299 12456500

[4] Wang X Maretti-Mira AC Wang L DeLeve LD. Liver-selective MMP-9 inhibition in the rat eliminates ischemia-reperfusion injury and accelerates liver regeneration. Hepatology. (2019) 69:314–28. 10.1002/hep.30169 30019419 PMC6325019

[5] Lin K Chen W Chuang H Lin S Chiang C Liang H. Idiopathic portal hypertension associated with POEMS syndrome mimicking liver cirrhosis in a patient with chronic HBV infection. Adv Dig Med. (2021) 8:115–20. 10.1002/aid2.13204

[6] Fan H Yan W Liu J Du C Xu Y Deng S. Analysis of misdiagnosis and missed diagnosis of POEMS with respect to hospital visit patterns. Chin J Clin Oncol. (2021) 48:1120–4.

[7] Plasma Cell Disease Group, Chinese Society of Hematology, Chinese Medical Association; Chinese Myeloma Committee-Chinese Hematology Association. Chinese expert consensus on the diagnosis and treatment of POEMS syndrome (2025). Zhonghua Xue Ye Xue Za Zhi. (2025) 46:389–96. 40623896 10.3760/cma.j.cn121090-20241219-00578 PMC12268301

[8] Dammacco F Rubini G Ferrari C Vacca A Racanelli V. ^18^F-FDG PET/CT: a review of diagnostic and prognostic features in multiple myeloma and related disorders. Clin Exp Med. (2015) 15:1–18. 10.1007/s10238-014-0308-3 25218739

[9] Kansagra A Dispenzieri A Fraser R Estrada-Merly N Sidana S Nishihori T. Outcomes after autologous hematopoietic cell transplantation in POEMS syndrome and comparison with multiple myeloma. Blood Adv. (2022) 6:3991–5. 10.1182/bloodadvances.2022007218 35507742 PMC9278304

[10] D'Souza A Lacy M Gertz M Kumar S Buadi F Hayman S. Long-term outcomes after autologous stem cell transplantation for patients with POEMS syndrome (osteosclerotic myeloma): a single-center experience. Blood. (2012) 120:56–62. 10.1182/blood-2012-04-423178 22611150

[11] Chen Y Lin J Jiang X Zhou Q Zhang H. A POEMS syndrome patient with idiopathic non-cirrhotic portal hypertension received the transjugular intrahepatic portosystemic shunt: a case report and literature review. Niger J Clin Pract. (2022) 25:1939–44. 10.4103/njcp.njcp_360_22 36412305

[12] Xu X Jing C Zhu T Jiang M Fu Y Xie F. Case report: POEMS syndrome with portal hypertension. Front Med (Lausanne). (2024) 11:1373397. 10.3389/fmed.2024.1373397 39109224 PMC11300253

[13] Campos S Agostinho C Cipriano MA POEMS. syndrome and idiopathic portal hypertension: a possible association. Rev Esp Enferm Dig. (2017) 109:393. 10.17235/reed.2017.4623/2016 28247771

[14] Wu L Li Y Yao F Lu C Li J Zhou W. Portal hypertension as the initial manifestation of POEMS syndrome: a case report. BMC Hematol. (2017) 17:9. 10.1186/s12878-017-0078-8 28503308 PMC5425989

[15] Belabbes F Houda Y Al Bouzidi A Bennani Y Ahnach M. Polyneuropathy, organomegaly, endocrinopathy, M-protein, and skin changes (POEMS) syndrome and idiopathic portal hypertension: a rare association. Cureus. (2022) 14:e24923. 10.7759/cureus.24923 35698702 PMC9187135

[16] Inoue R Nakazawa A Tsukada N Katoh Y Nagao T Nakanuma Y. POEMS syndrome with idiopathic portal hypertension: autopsy case and review of the literature. Pathol Int. (2010) 60:316–20. 10.1111/j.1440-1827.2009.02513.x 20403034

[17] Cui RT Yu SY Huang XS Zhang JT Li F Pu CQ. The characteristics of ascites in patients with POEMS syndrome. Ann Hematol. (2013) 92:1661–4. 10.1007/s00277-013-1829-7 23811954

[18] Stepani P Courouble Y Postel P Mezieres P Tossou H Couvelard A. Hypertension portale et ascite neutrocytique au cours du syndrome POEMS [Portal hypertension and neutrocytic ascites in POEMS syndrome]. Gastroenterol Clin Biol. (1998) 22:1095–7. 10051986

[19] Paciocco G Bossone E Erba H Rubenfire M. Reversible pulmonary hypertension in POEMS syndrome another etiology of triggered pulmonary vasculopathy? Can J Cardiol. (2000) 16:1007–12. 10978936

[20] Ferronato M De Molo C Bakken SM Leoni FG Vizioli L Di Donato R. Porto-sinusoidal vascular disorder, report of a novel association with POEMS syndrome. Future challenge for the hepatologist. Clin Res Hepatol Gastroenterol. (2023) 47:102126. 10.1016/j.clinre.2023.102126 37068710

