# Supplementary material for: POEMS syndrome complicated by portal hypertension resembling decompensated cirrhosis: a case report and diagnostic insights
Source: Front Med (Lausanne). 2025 Nov 3;12:1654338. doi: 10.3389/fmed.2025.1654338 (PMC12620467; doi:10.3389/fmed.2025.1654338)
Supplement: Supplementary file 1 [file Table_1.DOCX]

The latest follow-up data are as follows (Sept. 15, 2025):

Table S1 Serum VEGF levels after autologous stem cell transplantation 5 months

|  | Status | Reference Range |
| --- | --- | --- |
| VEGF pg/mL | 77.87 pg/ml | 0.00-160.00 pg/ml |


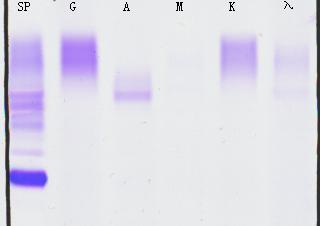


Figure S1 Immunofixation electrophoresis for IgAλ type weakly positive

Table S2 Quantitative detection of immunoglobulin on September 15,2025

| Immunoglobulin | Results and units of determination | Reference ranges |
| --- | --- | --- |
| Immunoglobulin IgG（IgG) | 10.60 g/L | 7.51-15.60 g/L |
| Immunoglobulin IgA（IgA) | 2.46 g/L | 0.70-4.00 g/L |
| Immunoglobulin IgM（IgM) | 0.70 g/L | 0.46-3.04 g/L |

Table S3 The levels of other biomarkers on September 15,2025

| Item | Results and units of determination | Reference ranges |
| --- | --- | --- |
| Creatinine（sCr) | 47.0 umol/L | 41.0-73.0 umol/L |
| uric acid（sUA) | 327.8 umol/L | 150.0-360.0 umol/L |
| Total protein（TP) | 64.0 g/L | 65.0-85.0 g/L |
| Albumin（ALB) | 40.0 g/L | 40.0-55.0 g/L |
| Alanine aminotransferase（ALT) | 54 U/L | 7-40 U/L |
| Aspartate amino transferase（AST) | 35 U/L | 13-35 U/L |
| Total bilirubin（TBIL) | 17.9 umol/L | 0.0-21.0 umol/L |
| G. F. R.（CKD-EPI) | 119.9 ml/(min*1.73m^2^) | 80.0-300.0 ml/(min*1.73m^2^) |
| Red-cell count（RBC) | 3.49 10^12^/L | 3.80-5.10 10^12^/L |
| hemoglobin（HGB) | 112 g/L | 115-150 g/L |
| Leucocyte count（WBC) | 2.82 10^9^/L | 3.50-9.50 10^9^/L |
| Total platelet count（PLT) | 104 10^9^/L | 100-300 10^9^/L |
